# Challenges and controversies of complex interventions in osteoarthritis management: recognizing inappropriate and discordant care

**DOI:** 10.1093/rheumatology/key062

**Published:** 2018-04-17

**Authors:** Krysia S Dziedzic, Kelli D Allen

**Affiliations:** 1 Institute for Primary Care and Health Sciences, Arthritis Research UK Primary Care Centre, Keele University, Keele, UK; 2 Thurston Arthritis Research Center, University of North Carolina, Chapel Hill, NC, USA; 3 Department of Veterans Affairs Health Care System, Center for Health Services Research in Primary Care, Durham, NC, USA

**Keywords:** controversies, challenges, osteoarthritis management, roles, Rheumatology, models of care

## Abstract

A number of controversies and challenges exist for the management of OA in health care. This paper describes the challenges and gaps in OA care, particularly in relation to population health management, complex interventions and outcomes. It sets this in the context of competing health priorities and multimorbidity, access to high quality conservative care, non-pharmacological therapies, resource limitations and models of care. The overuse of some therapies and neglect of others are discussed, as well as the potential for self-management. The roles of patient and public involvement and the healthcare team are highlighted in enhancing best care for OA and providing solutions for closing the evidence-to-practice gap. Implementation of models of care offer one solution to the challenges and progress of such implementation is described. Areas for further research are highlighted.

Rheumatology key messages

- There are significant evidence-to-practice gaps in osteoarthritis care.
  Algorithms and models of care are available as examples for rheumatologists and clinics to standardized osteoarthritis management.
- The healthcare team can play key leadership roles in musculoskeletal education and championing excellent osteoarthritis management.

## Introduction

The Bone and Joint Decade (2000–10) initiated the first international drive to prioritize OA and joint pain in older adults and its impact on the Western world [1, 2]. The diagnostic label of OA is ranked 11th in terms of its impact on years lived with disability. However, joint pain in those of 45 years and over is ranked as the number one cause of years with disability worldwide. According to health leaders and epidemiologists, the population trends are clear for the next 30 years, and with the ageing population and increased obesity and physical inactivity, joint pain and OA are set to rise.

Joint pain and OA are predominantly managed in primary care, and while there are national and international guidelines for the care and management of OA in adults [such as the National Institute of Health and Care Excellence (NICE), EULAR, Osteoarthritis Research Society International, ACR), there is a gap between what we know and what we do [e.g. 3–5]. This paper describes the challenges and gaps in OA care, particularly in relation to population health management, interventions and outcomes. It is set in the context of competing health priorities and multimorbidity, access to high quality care, resource limitations and models of care. The overuse of some therapies and neglect of others will be discussed, as well as the potential for self-management. Many of the challenges are predominantly taken from experiences in the National Health Service (NHS), UK. The role of the healthcare team will be highlighted in enhancing best care for OA and providing solutions for closing the evidence practice gap.

## Challenges and gaps in care

### Controversies in care

Data from a systematic review and meta-synthesis of qualitative studies exploring barriers and facilitators to offering best OA care, for example guideline recommendations for OA, identified only barriers and no enablers [6]. The findings addressed system-related barriers, disease-related barriers and patient-related barriers from which four distinct themes emerged from eight studies [6]: OA is not that serious, clinicians perceive they are under-prepared, to personal beliefs (e.g. negativity about OA), and dissonance in patient expectations. Such findings begin to explain some of the challenges in offering the range of treatment options recommended and described in the following sections.

#### Making and giving a diagnosis of OA

In primary care adults 45 years and over consulting with joint pain and limitations in everyday activity are more likely to receive NICE recommendations if a diagnosis of OA has been recorded in the medical electronic record [7]. Delay in diagnosis can lead to inappropriate treatment and suboptimal care. Overuse of imaging has prompted NICE to highlight the recommendation for diagnosing OA on clinical grounds and set a quality standard to audit this approach [8–10].

Diagnosis and subsequent treatment are often focused on a single, most painful joint rather than multisite joint problems [11]. Yet multisite joint pain is the most common presentation in consultations in primary care, and having more joint sites affected leads to more health care consultations irrespective of specialty or site [11]. As summarized in a recent systematic review, there is a paucity of evidence to guide the practitioner regarding treatment of multisite joint pain, with few studies describing interventions for considering OA in all affected joints [12]. Comorbidities also often go under-recognized in primary care and the community [13–16], which further hampers the holistic assessment recommended by NICE [8–10].

Reports have highlighted the impact of language when giving the diagnosis of OA. Health Care Professionals’ views can be perceived to be negative, for example, ‘nothing can be done’ and ‘it’s your age’ [17–20]. Unhelpful descriptions and terminology can easily transfer from the X-ray report into the consultation, with people with OA concerned for their ‘degenerative meniscal tear’. Use of language to talk about OA that offers more positive and supportive messages, such as ‘wear’ and ‘remodelling’, can enhance understanding of prognosis when verbal messages are backed up by written patient information [21]. Explaining that OA is part of a process of *repair* rather than degeneration can introduce a sense of optimism and reassurance because it offers a more positive outlook to life with OA, and can change patients’ and carers’ perceptions that OA inevitably leads to persistent pain, disability and joint replacement [21].

#### Self-management

**Fig. 1:**
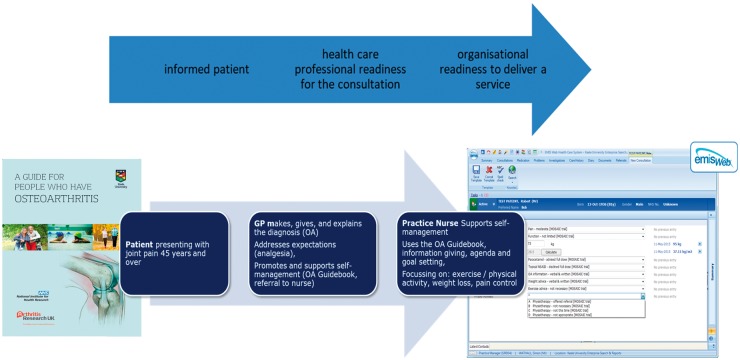
A model OA consultation developed using the WISE model and tested in the MOSAICS studyMOSAICS: Managing OSteoArthritis in ConsultationS.

One of the most complex interventions for the core management OA is self-management support. Delivering this well through a systematic, consistent approach across the pathway is a challenge. Ways to enhance self-management support within consultations for OA have been studied [22]. A Whole Systems Informing Self-Management Engagement model [23] for guided self-management of OA, including provision of patient information (e.g. OA guidebook) [24], care responsive to patient needs [25] and good access to follow-up care (practice nurse consultations), has been proposed [26] (see Fig. 1).

There is still limited knowledge on how to join up a system of care so that Quality Standards are delivered and assessed at each point in the pathway and the roles of the patient and carer are maximized. Patients with OA are clear that they want help and support to self-manage their condition, and that they want this to be health care professional-led [27–31]. Short-term changes are often not maintained in the long term, particularly for self-management programmes that require sustained lifestyle and behaviour changes [32–37].

The beneficial effects found in systematic reviews of OA self-management programmes, while small, demonstrate the need for ongoing self-management support throughout the course of the disease [38–40].

The use of digital platforms to enhance service delivery has been variable in health settings, but concerted efforts to redesigned musculoskeletal pathways and commission the use of digital platforms to enable patient information to transfer across systems and organisations are growing.

#### Exercise and physical activity

Since 2002 we have known the benefits of exercise as analgesia for OA pain [41, 42], but despite the substantial evidence for clinical and cost effectiveness of exercise, research funders still invest in underpowered studies looking at exercise *vs* no exercise [41]. More attention is needed on adherence to exercise, which is a major obstacle in exercise programmes. Currently the OA Trial Bank [43] is supporting work to determine subgroups who may respond better to exercise [44]. As of 2002 sufficient evidence had accumulated to show significant benefit of exercise over no exercise in patients with OA, and further trials were deemed unlikely to overturn this result [41]. However, the review highlighted that studies continued to be funded and published after this date. Furthermore, additional studies continue to be underpowered; in a Cochrane review, 35% of eligible studies recruited fewer than 25 participants in one or both allocation groups [45].

##### Topical NSAIDs and paracetamol

Topical NSAIDs have retained their role as the first line analgesia for peripheral joint OA, and electronic templates in general practice are known to enhance their uptake [7]. The role of paracetamol as first line analgesia has diminished following the evidence of poorer efficacy and increased side effects [46], but despite the concerns over paracetamol clinicians are unaware of the benefits of other therapies. Stopping ineffective therapies presents an implementation challenge. Clinicians continue with paracetamol when an alternative pharmacological or non-pharmacological approach such as topical NSAIDs and exercise might offer similar analgesic effects with fewer side effects [7, 37].

##### Opioids

Opioids are frequently used for OA pain [7]. Long-term opioids may benefit those with chronic pain but they have been shown to have adverse effects. In the UK there is evidence of an increase in prescribing of the more potent controlled and long-acting, long-term opioids [47]. Bedson *et al.* [47] showed that whilst primary care physicians had acted on national guidelines to reduce their use of new opioids, in cases in which opioids were already being prescribed, the shift towards using the more potent controlled and long-acting opioids continued. Those on more potent controlled opioids, either short-acting or long-acting, are of the greatest concern in relation to prescription opioid drug abuse and addiction [47].

#### Surgical approaches

Surgical treatments are offered for progressive pain and disability. The benefits of arthroscopic surgery on quality of life over the long term are minimal, and those with knee disease experience very small improvements in pain and function when compared with others who receive conservative management [48]. As the evidence fails to support a persistence of benefit over the long term, there is a trade-off between the marginal short-term benefits against the burden of the surgical procedure [48]. Many international guidelines do not recommend arthroscopy unless there is true mechanical locking of the knee [e.g. 8, 49]. The use of arthroscopy for knee OA has decreased; however, it is still prevalent [50]. Pressure from patients to do something, the perception that other options are limited, that surgeons want to meet patients’ expectations, and time pressures in clinic, all appear to influence the choice to undertake arthroscopic procedures [50]. Winter *et al.* [51] evaluated the risk of total knee replacement (TKA) in patients undergoing knee arthroscopy. They found that those undergoing arthroscopy might anticipate an annual rate of TKA in the order of 2%, with higher rates among older patients and those with more advanced OA [51]. Clinicians and patients considering knee arthroscopy should discuss the likelihood of subsequent TKA as they weigh risks and benefits of surgery.

Knee OA can be managed well non-surgically, but many patients and providers still consider total joint arthroplasty (TJA) the only option, especially at later stages of disease [50, 52]. Many patients have good results with TJA [49], but there is still a subset who have sub-optimal results, and all the factors that predict good outcome are unknown. Data from the Osteoarthritis Initiative on patients who had undergone TKA were used to determine the prevalence rates of TKA surgery classified as appropriate, inconclusive and inappropriate [53]. Approximately one-third of TKA surgeries were judged to be inappropriate.

Surgeons have recognized the need for support tools for making the decision for TJA [54]. Canadian stakeholders have identified several potential criteria for TJA: evidence of arthritis on joint examination; patient-reported symptoms negatively impacting quality of life; an adequate trial of appropriate non-surgical treatment; realistic patient expectations of surgery; mental and physical readiness of the individual for surgery; and patient-surgeon agreement that potential benefits exceed risks [55]. However, there remains a need for validated tools to adequately assess and communicate appropriateness criteria for TJA.

For patients with no previous history of knee repair surgery and with very minimal OA changes, autologous chondrocyte implantation may offer a treatment option for those with persistent symptoms after conservative therapy and with cartilage defects over 2 cm^2^ [56]. However, there remains a need for evidence on long-term effectiveness of this procedure.

### Training gap

Consensus work and surveys highlight the need for health care professionals’ training in the skills of making and giving the diagnosis of OA, supporting self-management and delivering care in line with international guideline recommendations for OA [e.g. 57]. Audit of educational needs of health care professionals and patients shows the mismatch between educational need and training delivered [58]. Education and training packages for primary health care professionals now offer accredited online musculoskeletal modules [e.g. 59, 60].

Maximizing the use of transferable skills by health care professionals has been neglected. For example, general practice nurses have expertise in running chronic disease clinics, and many of the techniques for supporting self-management, for example, keeping active and weight management, can be used across long term conditions. Unfortunately, general practice nurses are given few training opportunities to enhance their skills in supporting self-management for OA.

### Outcomes of care

The whole research cycle takes research from priority setting right through to implementation of best evidence. Whether the same outcomes used to demonstrate clinical effectiveness in trials are the same as those needed for evaluation of services remains unclear. Allen *et al.* [4] in their evaluation of models of care listed commonly used outcomes that included disease-specific measures for OA as well as generic health measures and OA quality indicators of care [e.g. 61, 62]. New measures, such as the Musculoskeletal Health Questionnaire (MSK-HQ), may also be useful for assessing outcomes of care [63] and offer an opportunity to embed outcome measures in routinely recorded medical record data. Big data and aggregated, anonymized medical record data will provide the means for understanding variations in care at an organization level [e.g. 64]. The challenges of information governance, consent, anonymization and aggregation of data are very difficult issues to resolve and are often tackled only at a local level.

### Closing the evidence-to-practice gap

Given the current challenges in offering guideline recommendations, closing the evidence-to-practice gap is key. From 2002 the Arthritis and Musculoskeletal Alliance in the UK developed standards of care for OA, building on what a person with OA should expect to receive but their implementation has been lacking [65]. With the advent of NICE OA guidelines [8, 9] and NICE Quality Standards [10] we now have a set of recommendations that can be adopted, but as yet there has been no UK audit of these.

These gaps in knowledge are recognized, and yet closing the gap is complex [66]. The evidence we produce regarding how to deliver best care has its own limitations. The individual studies themselves can be methodologically outstanding and serve to increase knowledge, but transferring this knowledge to real world settings is difficult. Evidence underpinning the recommendations for clinical guidelines is predominantly derived from studies of knee OA, with fewer studies for the hip and even fewer for the hand and foot. Single treatment approaches are often studied in isolation and there is a lack of studies of integrated packages of care.

Lau *et al.* [66] described the causes of the evidence-to-practice gap in primary care and the ways in which the evidence gap could be addressed, although even the most effective interventions such as clinical opinion leaders show at best only small effects, and there is no certainty that multiple approaches work better [66]. What Lau and colleagues did highlight was the importance of context and the role of organizations in influencing the uptake of best practice. Context means policy such as NICE guidelines, public awareness of OA and its care, economic climate and funding, stakeholder buy-in (e.g. Sustainability Transformation Partnerships), technological advances and infrastructure to deliver best care.

For health services, Clinical Networks can offer an approach to knowledge mobilization between ‘what we know’ and ‘what we do’. The National Clinical Director for Long Term Conditions with NHS England and the Arthritis and Musculoskeletal Alliance have made musculoskeletal health a priority across the four key regions in England with the development of musculoskeletal knowledge networks to embody the potential for sharing models of care and good practice with trusted partners within a geographic boundary [67].

## OA care models and pathways to enhance coordination

Many of the specific gaps in OA care can be at least partly attributed to a lack of care coordination and a purposeful management approach. Without a model for how, when and by whom specific OA-related therapies are provided, there is a high risk that some components of care will be neglected. This can be particularly challenging since OA treatments are delivered by different types of providers, yet a point person is not always apparent. Another challenge is that although OA treatment guidelines provide information about therapies that should be delivered, they are largely silent regarding when specific treatments are appropriate and how various therapies may best fit together in a comprehensive treatment approach. Unfortunately, research to date has provided little evidence regarding the optimal timing, integration or criteria for different OA therapies. However, a number of efforts have applied practical, clinical experience to OA treatment guidelines, developing treatment algorithms and care models that can serve as guides and examples for rheumatologists and other clinicians.

### Development of OA treatment algorithms

**Fig. 2:**
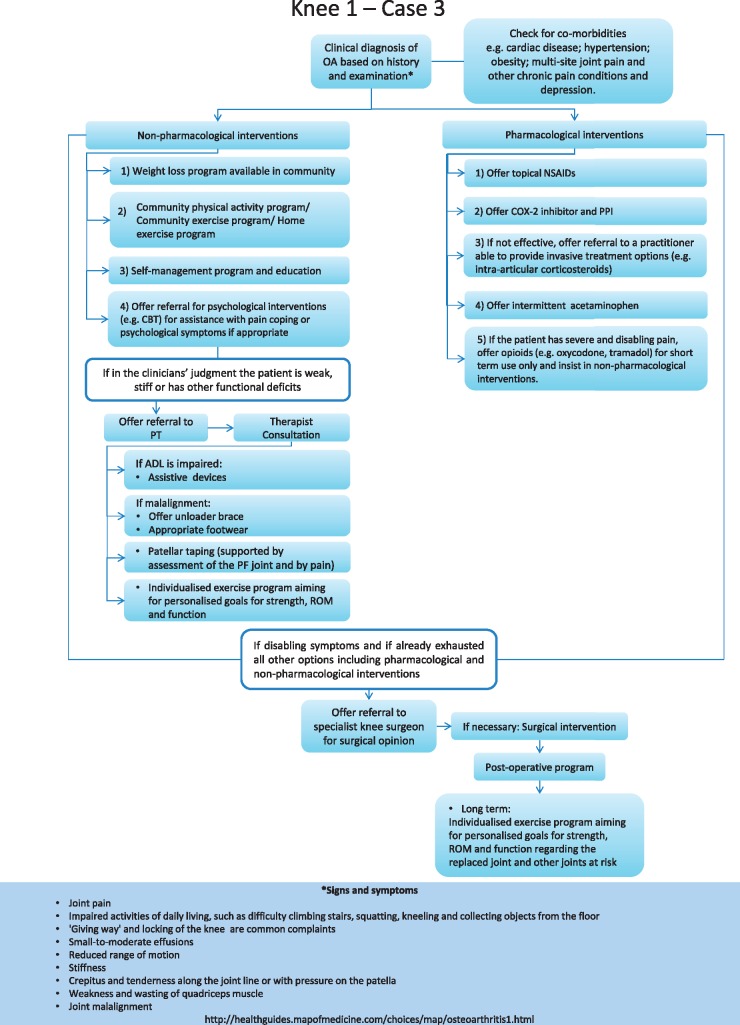
Clinical algorithms for OA treatment of the knee with several co-morbidities Reprinted from Osteoarthritis and Cartilage, Volume 24, Meneses SR, Goode AP, Nelson AE, Lin J, Jordan JM, Allen KD *et al.* Clinical algorithms to aid osteoarthritis guideline dissemination, Pages 1487–99, Copyright 2016, with permission from the Osteoarthritis Research Society International, published by Elsevier Ltd [69].

Two recent international efforts developed clinical algorithms for OA treatment [68–70]. Meneses *et al.* [69] performed a systematic review of OA treatment guidelines and then used an iterative expert panel process to derive clinical algorithms for the treatment of hand, hip and knee OA. These algorithms consider key issues such as comorbid health conditions and offer a step-wise approach for delivery of pharmacological and non-pharmacological therapies. The algorithms also provide general decision rules for when more intensive or different therapies should be considered. An example algorithm for a patient with knee OA and several comorbidities is shown in Fig. 2; four different algorithms are available and can provide a practical approach for rheumatologists and clinics to operationalize treatment of OA. Bruyére and colleagues [68] also developed algorithms that focus on pharmacotherapy for knee OA.

### Example models of OA care

There have also been efforts internationally to develop models for delivering recommended OA therapies within health systems. A selection of these models is described briefly here and in greater detail elsewhere [4]. These models vary in terms of the range of OA treatments included (e.g. some focusing on a specific area such as weight management or physical activity and some with a more comprehensive approach), types of providers involved and reimbursement model. Therefore, these programmes provide a range of examples that rheumatologists can consider with respect to feasibility of implementation in their clinical context.

#### Amsterdam Osteoarthritis Cohort—Netherlands

Individuals from the Amsterdam Osteoarthritis Cohort are eligible for an OA management programme if they have hip and/or knee OA, and if pain is non-traumatic, sufficient to seek care and attributed by a clinician to a hip or knee joint. This programme offers coordinated multidisciplinary management that includes supervised exercise according to a knee joint stabilization programme [71], occupational therapy, psychological support and medical management. Funding for this programme is from various sources, including the health care system and trials conducted within the cohort.

#### Better management of patients with OA—Sweden

Better Management of Patients with Osteoarthritis [72] is a programme for individuals with hip, knee, hand or shoulder OA [73] who have non-traumatic pain, sufficient to seek care and attributed by a clinician to their joint. In this programme physiotherapists, occupational therapists and expert patients (OA-communicators) provide education, self-management support, exercise recommendations, an optional individualized exercise programme and optional supervised exercise group sessions.

There is also an online version, Joint Academy [74], which includes recommended exercises, interactive lessons, reports and tracking tools. In addition, patients can communicate with a physical therapist in the context of the online programme. Participants from anywhere in the world can sign up for Joint Academy, and the current cost for the 6-week core programme is $45USD.

#### Enabling Self-management and Coping with Arthritic Pain Using Exercise—UK

Enabling Self-management and Coping with Arthritic Pain using Exercise (ESCAPE-pain) [75] is a rehabilitation programme for people with joint pain, including OA [42]. It integrates self-management and coping strategies with and individualized exercise programme. ESCAPE-pain is typically delivered by physiotherapists but can be administered by other qualified healthcare workers in various settings. The delivery format is in small groups, with meetings twice per week for 6 weeks. There is also an ESCAPE-pain app that mirrors the in-person programme.

#### Good Life with Arthritis in Denmark

Good Life with Arthritis in Denmark (GLA:D) is programme for individuals with hip and/or knee OA; similar to Better Management of Patients with Osteoarthritis, patients must have non-traumatic pain sufficient to seek care and attributed by a clinician to OA of the hip or knee joint. GLA: D focuses on self-management and exercise components of OA treatment [76]. There are three patient education sessions provided over the course of 2 weeks; the first two sessions are delivered by a physiotherapist and the third by an expert patient who previously participated in GLA:D. These education sessions are followed by 12 sessions of supervised neuromuscular exercise sessions based on the NEuroMuscular Exercise programme [77]. GLA:D has now been disseminated in many countries, and more information (as well as contact information for the developers) can be found on the GLA: D website [78].

#### Joint Implementation of Osteoarthritis Guidelines—UK

Joint Implementation of Osteoarthritis Guidelines [79] is a comprehensive OA management programme based on evidence from the MOSAICS trial [26]. This programme is offered to individuals of 45 years and over who are consulting in general practice, have knee, hip, hand and/or foot OA and have joint pain that limits function. The programme is initiated with a model OA consultation with a general practitioner and a practice nurse, including making, giving and explaining the OA diagnosis, giving an OA guidebook, offering analgesia and referral to a practice nurse (Fig. 1). The practice nurse then provides up to four sessions supporting self-management; these include exercise and physical activity advice using Arthritis Research UK booklets, weight management and support for pain relief. An electronic template is used to measure key quality indicators of OA care within the programme. Further implementation of this model is being tested in the Netherlands, Norway, Denmark and Portugal (European Institute of Innovation & Technology (EIT)-Health funded Joint Implementation of oSteoArthritis Guidelines in Western Europe (JIGSAW-E )) [80].

#### Osteoarthritis Chronic Care Programme—Australia

Osteoarthritis Chronic Care Programme is a programme for patients with doctor-diagnosed knee and/or hip OA, along with pain in the affected joint on most days of the past month (pain visual analogue scale ⩾ 4 out of 10) [81, 82]. This programme involves multidisciplinary, individually tailored, physiotherapy-led OA management. Treatments include exercise, diet, psychological support, occupational therapy, orthotics and medical management. Patients can be referred to the programme by any health care provider, and it was initially funded through the public hospital system. (The Osteoarthritis Chronic Care Programme model of care and other relevant documents can be accessed at [83].)

#### Osteoarthritis Healthy Weight for Life—Australia

The Osteoarthritis Healthy Weight For Life programme [84] is offered to individuals with knee or hip OA diagnosed by radiological evidence who are overweight (BMI ⩾ 28) and have significant joint symptoms [85]. This 18-week programme focuses on behavioural aspects of OA management, including weight loss and improved nutrition, a physical activity plan and physiotherapist-delivered exercises (strength, balance and mobility), personalized online symptom, progress and satisfaction tracking (with phone or mail options) and personal motivation via phone or other tools. This programme is available in Australia at no charge via some health insurance providers, but for those without private health insurance (a substantial proportion of the population) there is a cost involved.

## Roles of the multidisciplinary team

The multidisciplinary team of health care professionals, for example, rheumatology nurse, physiotherapist, community pharmacist, dietician and rheumatologist, can assist with transferable skills and increase confidence in primary care in delivering quality care, reduce overuse of X-ray in the diagnosis of OA, reduce inappropriate referral to orthopaedic surgery and increase the uptake of core non-pharmacological treatment with confidence in the safety of exercise and its use as an analgesic [86].

## The role of rheumatologist in OA management

OA is most often managed in primary care, with referral to secondary care typically only in more advanced stages or complex presentations. However, rheumatologists have a key role and opportunity for leadership in OA management [59]. First, rheumatologists have content expertise in the management of OA and can therefore be leaders in health systems, driving appropriate models of care and quality improvement. Leadership in this area is greatly needed, considering the gaps in quality of OA care and often a lack of a champion for treatment of this health condition. Second, OA commonly co-occurs with other rheumatic conditions, particularly among older adults. Therefore, rheumatologists can set an example of delivering the highest quality of OA care, incorporating both pharmacological and non-pharmacological therapies. The following are specific practical recommendations for ways rheumatologist can lead the way in optimizing OA care:

### Provide education and support in OA management to primary care colleagues

Because primary care providers are responsible for managing a wide range of health conditions, it is not realistic to expect they will typically be experts in musculoskeletal medicine. Rheumatologists can bridge this gap by providing periodic educational sessions on OA care for primary care providers, covering evidence-based therapies and specific challenging clinical situations. In some health care settings, individual rheumatologists or groups of rheumatology clinicians may be able to provide virtual or electronic consults to provide input on specific patients or scenarios. This may be particularly useful for rural primary care providers who do not have ready in-person access to musculoskeletal expertise.

### Provide leadership in developing an OA patient pathway or model of care within the healthcare system

The example algorithms and models of care described above can be an excellent starting place for developing a context-appropriate OA pathway. Because of content area expertise, rheumatologists are equipped to lead multidisciplinary efforts (involving primary care, orthopaedics and rehabilitation) to develop and implement pathways that facilitate evidence-based and comprehensive OA care.

### Connect with community organizations and resources

Behavioural treatments such as exercise and weight loss are key components of managing OA. Resources to support patients in these behaviours are often not available within the healthcare system. However, many services are available within the community. Rheumatologists can have a significant impact on patients’ OA management by connecting them with evidence-based, reputable community resources to support healthy behaviours.

## Involving the patients and the public

Rheumatology has developed a successful track record of involving patients and the public in shaping OA care pathways. Patients and the public have an increasing and influential role in shaping services and supporting changes in practice in primary care, and their role in secondary care has been established for some time.

## Key areas for future research

Algorithms and models of care are available as examples for rheumatologists and clinics to standardize OA management and the healthcare team can play key leadership roles in musculoskeletal education and championing excellent OA management. However, there are significant evidence-to-practice gaps in OA care. The following are research areas and methodological considerations that would significantly improve the evidence base underlying optimal OA management: research participants should represent the range of patients seen for OA, including those with multi-joint disease, those with multiple comorbidities and the oldest old. This will enhance the generalizability of findings so that they can inform real-world clinical practices; studies should systematically examine heterogeneity of treatment effects to identify patient characteristics that predict response to therapies. This will help to inform tailoring of treatment regimens in clinical settings; although studies of individual treatments or interventions are still needed in some areas, there is a great need to study more novel, complex and integrated approaches to OA management that mirror clinical scenarios, consider the whole person and engage with caregivers and other support systems; there is a need for a greater focus on implementation research in OA, identifying models of care that can be successfully delivered, as well as considerations for cost effectiveness.
